# Loss of Function of the Mouse *Sharpin* Gene Results in Peyer’s Patch Regression

**DOI:** 10.1371/journal.pone.0055224

**Published:** 2013-02-12

**Authors:** Rosemarie Seymour, Bobbi-Jo Shirley, Harm HogenEsch, Leonard D. Shultz, John P. Sundberg

**Affiliations:** 1 The Jackson Laboratory, Bar Harbor, Maine, United States of America; 2 Department of Comparative Pathobiology, Purdue University, West Lafayette, Indiana, United States of America; University of California, San Francisco, United States of America

## Abstract

Peyer’s patches (PP) are an important component in the immune response against intestinal pathogens. Two independent, spontaneous mutations in the mouse *Sharpin* gene (*Sharpin^cpdm^* and *Sharpin^cpdm-Dem^*) result in the absence of PP and disrupted splenic white pulp in adult mice, although a full complement of lymph nodes is present. Here we report that rudimentary PP begin to develop in *Sharpin^cpdm^* mice during embryogenesis, but lack the organizational patterns that are typical of this tissue. In the present study, small intestines examined at weekly intervals from birth to maturity showed spontaneous regression of PP in mutant mice with concurrent infiltration of granulocytes. At 5 to 6 weeks of age, only indistinct remnants of granulocytic accumulations remain. Transplantation of normal bone marrow into *Sharpin^cpdm^* mice at 7 days of age did not prevent regression of PP in bone marrow chimeras examined at 7 to 8 weeks of age. These findings indicate that SHARPIN expression is required for the normal development and maintenance, but not initiation, of PP.

## Introduction

Peyer’s patches (PP) are organized secondary lymphoid tissues located in the anti-mesenteric wall of the small intestine of mammals. They comprise the major inductive sites for adaptive immune responses to intestinal antigens. PP are an important source of IgA-producing B cells that develop through class switching from IgM^+^ cells under the influence of T cells in the germinal centers of PP, although T cell–independent IgA production also occurs [1].

PP development (reviewed in [2]) is a nuclear factor kappa B (NFKB)-dependent process initiated in the intestinal stroma of embryonic mice by vascular cell adhesion molecule 1 (VCAM1)^+^, intracellular adhesion molecule 1 (ICAM1)^+^ stromal organizer cells on the antimesenteric surface of the small intestine at 15.5 days post conception (dpc), followed by recruitment of lymphoid tissue inducer (LTi) cells. LTi cells express multiple surface markers, including CD4, PTPRC (CD45R or B220), IL7R, and CXCR5 (BLR1). Interactions between IL7R signal-induced lymphotoxin (LT) alpha1beta2 expression on LTi cells and the LT beta receptor (LTBR) on organizer cells activate NFKB transcription factors, driving chemokine expression to create the microenvironment for further PP development. Compartmentalization of PP anlagen into biologically efficient structures is initiated near the end of gestation in mice, prior to the entry of lymphocytes. Recruitment of mature B and T cells begins after 18.5 days post conception (dpc), and segregation of B and T cell compartments is completed postpartum [3].

C57BL/KaLawRij*-Sharpin^cpdm^*/RijSunJ mutant mice have severe, progressive multiorgan inflammation [4]. PP are absent in adult (examined at 6+ weeks of age) *Sharpin^cpdm^* mice, as determined by serial sections of Swiss rolls of the small intestine, and accordingly fecal IgA levels are greatly reduced [5]. The spleen has decreased white pulp, lacks a defined marginal zone, and B cell function is impaired, with significantly reduced serum IgG, IgA, and IgE. Although all lymph nodes are present, the remaining secondary immune organs lack follicular dendritic cells (FDCs), B cell follicles, and germinal centers [5], [6].

To begin to elucidate the mechanism underlying the absence of PP in adult *Sharpin^cpdm^* mutant mice, the small intestines of neonatal and young mice homozygous for the recessive *Sharpin^cpdm^* mutation were examined for evidence of PP anlagen. Surprisingly, PP populated by B and T lymphocytes were present in neonatal *Sharpin^cpdm^* mice, although they were smaller in size and lacked the organization seen in PP of mice heterozygous or wildtype for *Sharpin* (controls). Spontaneous regression of PP in maturing *Sharpin^cpdm^* mice was typically associated with infiltration by granulocytes.

## Results

**Figure 1:**
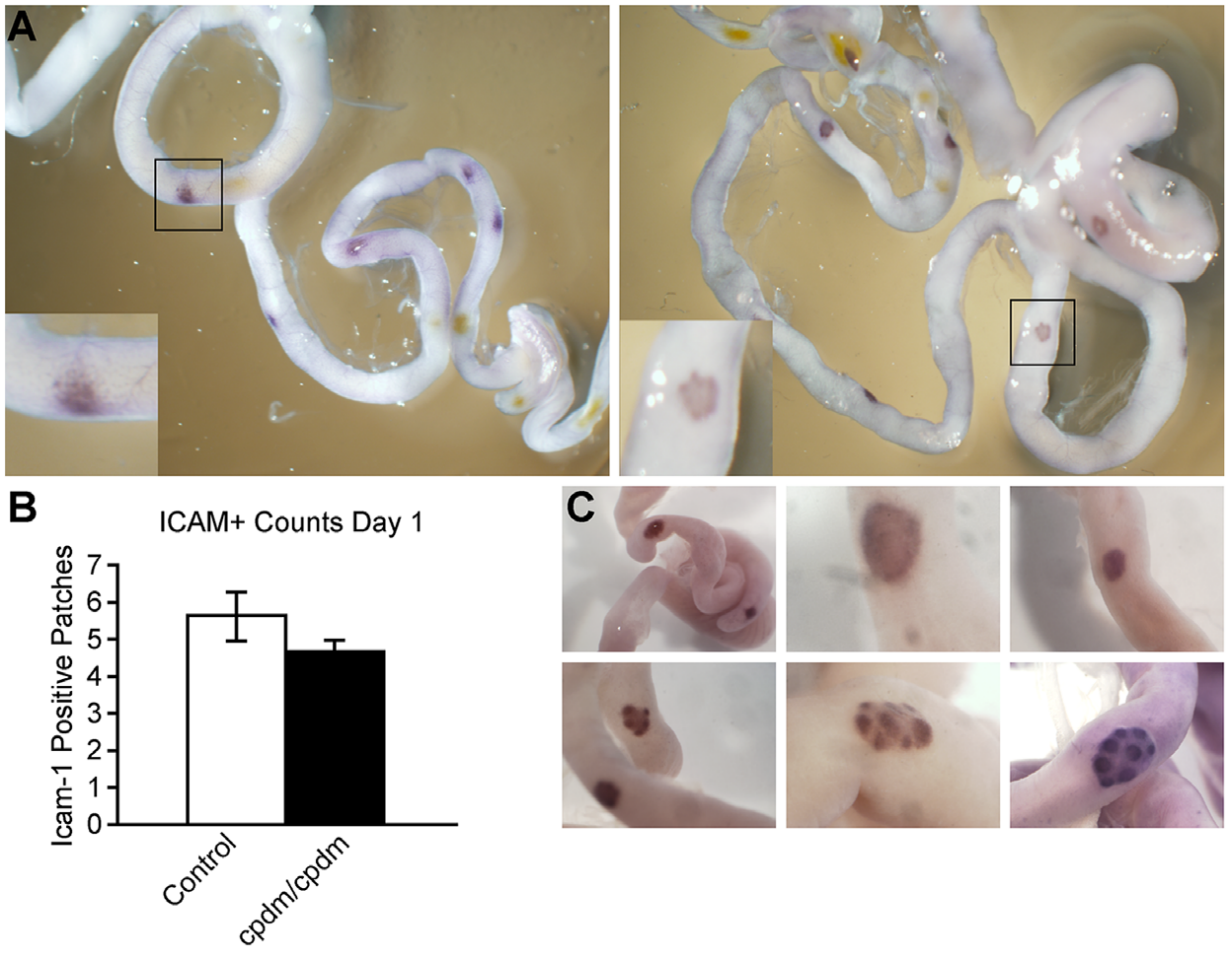
Lack of compartmentalized Peyer’s patches (PP) in *Sharpin^cpdm^* mutant mice. (A) Intercellular adhesion molecule (ICAM1) labeling in the small intestine of 1 day old mice. Numerous ICAM1^+^ patches are present in the *Sharpin^cpdm^* mutant mice (left), however they lack the distinct compartmentalization seen in most ICAM1^+^ patches of control (+/?) mice (right). (B) Mean ICAM1^+^ counts for a litter of 1 day old *Sharpin^cpdm^* mutant (N = 3) and control (+/?) mice (N = 8). Bars indicate standard error of the mean. (C) Anti-CD45RB (B220) labeling in the small intestine of mutant *Sharpin^cpdm^* (top panel) and control (bottom panel) mice at (left) 4 days, (center) 1 week, and (right) 2 weeks of age. Images are representative of 3 to 4 mice per group; PP of control mice appear compartmentalized and follicular structures are evident, and overall size of PP subjectively increase with age, while those of mutant mice do not show these features. WMI.

Clusters of ICAM1^+^ cells were observed by whole mount immunohistochemistry (WMI) in the small intestines of neonatal (0–1 day postpartum) mutant and control mice. There was no significant difference in the number of these patches (mean, 4.7 and 5.6 in mutants and controls, respectively), although increased labeling intensity and compartmentalization of these cells could be readily observed in PP of control, but not mutant, mice **(** **Fig. 1A, B** **)**.

B220-positive foci were readily identified at the anti-mesenteric surface of the small intestines of neonatal mice by WMI. At 1 to 2 weeks of age, multiple individual follicles could be discerned in the PP of control mice. The size of follicles and of individual PP subjectively increased with age in control mice. Representative findings are shown in **Fig. 1C**. B220-positive foci were also present in the intestine of mutant mice between 0 days and 5 weeks of age, however, these did not contain apparent follicular structures. They were less organized, smaller, and less prominent than those of the controls, and the intensity of the labeling became variable and overall decreased greatly by 3 weeks of age. Although organized PP could easily be identified and B220^+^ staining was still present in control PP up to 6 weeks of age this method became less reliable (with less uniform staining) in older mice.

**Figure 2:**
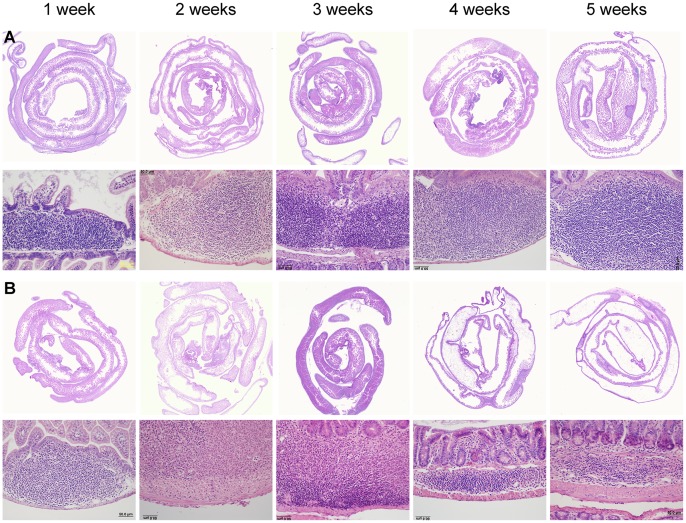
Regression of lymphoid tissue in *Sharpin^cpdm^* mutant mice. Histologic sections of Peyer’s patches in control mice (A) and residual lymphoid tissue in *Sharpin^cpdm^* mutant mice (B) at 1 to 5 weeks of age are shown. Images represent at least 3 mice per group examined at each time point. Some variability is observed in the cellular composition of individual lymphoid tissue remnants in mutant mice, however eosinophilic/granulocytic and stromal effacement is common. Lymphoid patches were not found in some mutant mice at 4 to 5 weeks using this method. H&E.

Sections of the small intestine were evaluated by microscopic examination of hematoxylin and eosin (H&E) stained Swiss rolls of the small intestine, confirming the presence of PP in young mutant and control mice. These patches did not have defined follicles in mutant mice, in contrast to controls. In some PP of mutant mice at 2 to 3 weeks of age, there were increased numbers of non-lymphoid cells, including granulocytes, stromal cells, and macrophages. Non-lymphoid cells comprised a small fraction of the cells in PP of the controls. At 4 and 5 weeks of age, a dramatic change was seen in the size and cell population of the few remaining patches in *Sharpin^cpdm^* mice, in that they were composed of sparse numbers of lymphocytes admixed with granulocytes, macrophages, and increased amounts of fibrous connective tissue (**Fig. 2**). In mutant mice aged 6 weeks and older, no distinct PP were seen using this method. However, occasional remnants of granulocytic accumulations were observed in the antimesenteric submucosa of the small intestine, presumably the sites of degenerate PP.

**Figure 3:**
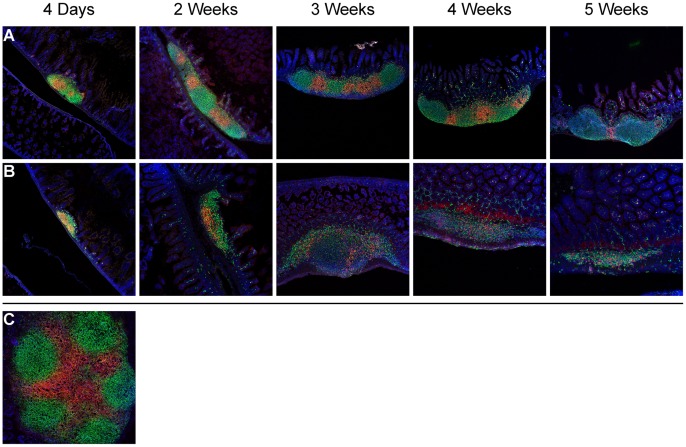
B220^+^ B and CD3^+^ T lymphocyte organization in Peyer’s patches (10X). Images represent at least 3 control mice (A) and *Sharpin^cpdm^* mutant mice (B) examined at each time point. Lymphoid patches were not found in the small intestines of some 4- and 5-week old *Sharpin^cpdm^* mutant mice using this method. (C) Longitudinal section of a Peyer’s patch from a control mouse showing normal segregation of B and T cell zones. Green–B220^+^ B cells, Red–CD3^+^ T cells, Blue–DAPI (nuclear) counterstain. IFA.

To determine the composition and organization of PP remnants in young mutant mice, frozen sections of the intestine were examined using indirect immunofluorescence (IFA) microscopy with antibodies against CD45RB and CD3 for B and T cells, respectively. Similar to results obtained via other methods, PP of mutant mice appeared smaller than the PP of controls at all time points, and fewer PP were typically found. In addition, mutant PP remnants were consistently less organized, with little compartmentalization of B and T cells and no follicular structures observed. As mice matured, increasing numbers of non-B, non-T cells were observed in the PP remnants of mutant mice, consistent with the infiltration of granulocytic populations observed by H&E staining. By contrast, the PP of control mice subjectively increased in size with age, and exhibited distinct compartments, with CD3^+^ T cells mostly concentrated in the interfollicular areas and occasionally seen in B cell follicles (**Fig. 3**).

**Figure 4:**
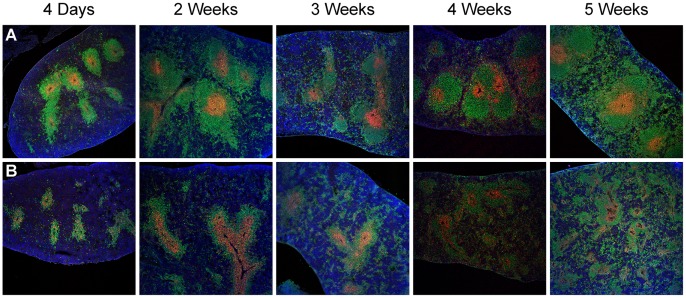
Comparison of splenic white pulp in *Sharpin^cpdm^* mutant and control mice. The white pulp of control (A) mice is discrete and well-organized. B and T cell regions of the *Sharpin^cpdm^* mutant (B) spleen are smaller, organization is poor, and splenomegaly is common (10×). Images represent at least 3 mice per group examined at each time point. Green–B220^+^ B cells, Red–CD3^+^ T cells, Blue–DAPI (nuclear) counterstain. IFA.

The splenic white pulp of adult *Sharpin^cpdm^* mutant mice is disorganized with poor separation of B and T cell areas [5]. To determine whether the observed changes in the PP of mutant mice parallel changes in the organization of white pulp, frozen sections of the spleen were examined in mutant and control mice at weekly intervals up to 5 weeks of age, identifying cell populations as described for the intestine. While differences were subtle in mice <2 weeks of age, the white pulp in mutant mice appeared smaller and more dispersed than that of controls at all time points. White pulp size and organization subjectively increased in the spleens of control mice as they matured. At 4 and 5 weeks of age, the white pulp of *Sharpin^cpdm^* mutant mice became progressively more disorganized with increased mixing of B and T cell populations and scattering of B cells along with increased numbers of non-lymphoid cells. Splenomegaly caused by increased extramedullary hematopoiesis was common in mutant mice. In control mice, segregation of the red and white pulp was more distinct, and the white pulp contained numerous lymphocytes. Representative images are shown in **Fig. 4**.

To further evaluate whether loss of PP may be attributable to defects in hematopoietic cells, stromal cells, or both, bone marrow cells from healthy histocompatible donor mice transgenically expressing green fluorescent protein (GFP)^+^ were transferred into irradiated *Sharpin^cpdm^* and control mice at 7 days of age. Recipient mice (6 mutants and 8 controls of both sexes) were euthanized and necropsied at 7 to 8 weeks of age. Engraftment of donor cells was confirmed by immunohistochemistry for GFP^+^ cells [7] in the spleen, liver, colon, and cecum. Although the skin phenotype of mutant mice was subjectively milder than expected (ie, mild to moderate rather than moderate to severe) in most recipents, no evidence of reconstitution of PP was detected histologically (by examination of Swiss rolls of the intestine as described) in any of the recipient mutant mice. Normal PPs were found in recipient control mice histologically, and repopulation of lymphoid tissues including the gut associated lymphoid tissues of the large intestine and cecum by donor cells was subsequently confirmed by immunohistochemistry for GFP^+^ cells.

## Discussion

The development of PP during late embryogenesis has been well characterized in recent years. This varies from development of other secondary lymphoid organs in some key ways. In lymph node organogenesis, early lymph sacs bud from vascular endothelium in a prospero-related homeobox 1 (PROX1)-dependent manner to accumulate LTi cells, while PP are initiated by the *Ret* proto-oncogene-dependent clustering of previously scattered LTi cells with KIT^+^, ITGAX (CD11C)^+^ initiator cells in the small intestine [8], [9]. The local microenvironments for these tissues require different cytokines and signaling pathways to induce lymphoid development. Whereas PP require JAK3-dependent IL7R signaling, lymph nodes require TRAF6-dependent TNFSF11 (TRANCE) signaling for the differentiation and function of LTi cells [10]–[12]. Additionally, the signaling requirements for cervical and mesenteric lymph nodes can vary from those of other lymph nodes (reviewed in [13]). Development of splenic white pulp is not dependent on LTi cells, but its organization and maturation requires activation of NFKB through LTA1B2 signaling in lymphocytes [14]. Similarly, although pathways involving TNF, TNFRSF1A, LTA, LTB, TNFSF14 (LIGHT), and the LTBR all have complex roles in the development and organization of secondary lymphoid organs [15]–[21], LTA1B2 activation of NFKB transcription factors via the LTBR is indispensible for lymph node and PP development. The LTBR is one of several tumor necrosis factor (TNF) superfamily receptors, the binding of which lead to activation of inhibitor of kappa B kinases (IKKs) and MAP3K14 (NFKB inducing kinase [NIK]); these phosphorylate inhibitory proteins that sequester NFKB homo- and heterodimers in the cytoplasm, and subsequent ubiquitin-proteasome pathway degradation of the inhibitors allows nuclear translocation of NFKB complexes for transcription of target genes (reviewed in [22]). Among genes downstream of the LTBR and other TNF superfamily receptors are those involved in inflammation, cell survival, and innate immunity (primarily through canonical activation of the NFKB1-P50 heterodimer), lymphoid tissue development (primarily through noncanonical activation of the NFKB2-P52 heterodimer), and organization of these structures (involving both pathways).

PP are absent in mice lacking *Lta*, *Ltb* [17], [18], *Ltbr* [21], *Map3k14* [23], *Il7r*, *Jak3* [10], *Rorc* [24], *Id2* [25], *Ikzf1* (*Ikaros*) [26], *Relb*, *Nfkb2* [27], or the *Ret* proto-oncogene [9]. Significant blunting of PP development is seen in mice lacking *Cxcl13* [28] or its receptor *Cxcr5* [29] and in some mice lacking *Tnf* [30], [31] or *Tnfrsf1a* [32]. It is interesting to note that in all of these mice, with the exception of those with defects in the IL7 receptor pathway, lymph node genesis is also affected. In contrast, adult *Sharpin^cpdm^* mutant mice have a full complement of lymph nodes, although these are often infiltrated by granulocytes, whereas they lack PP [5].

SHARPIN was recently discovered to be component of the RBCK1 (HOIL1L) and RNF31 (HOIP)-containing linear ubiquitin chain assembly complex. Linear ubiquitination of the IKK regulatory subunit IKBKG (NEMO) is among the functions of this complex, and SHARPIN deficiency was shown to result in impaired CD40- and TNFA-induced activation of the IKK complex and subsequent canonical NFKB activation in B cells and mouse embryo fibroblasts *in vitro* [33]–[35]. Although activation of the noncanonical NFKB pathway, which is essential for secondary lymphoid organ development, was not reported to be affected in B cells or mouse embryo fibroblasts, SHARPIN may potentially influence this pathway in other cell types or in specialized microenvironments during lymphoid tissue development. Investigators demonstrated that ablation of TNF in *Sharpin^cpdm^* mutant mice rescued the inflammatory skin phenotype and eliminated granulocyte accumulations in various organs, but did not rescue development of the splenic marginal zone or PP [33]. Thus, the inflammatory cell infiltrates observed in regressing PP of young mice in the present study are not likely to be the cause of PP loss. Dysregulation of local chemokine expression in high endothelial venules and stromal tissues may contribute to the accumulation of granulocytes in secondary lymphoid organs in this disease.

Findings of the present study demonstrate that while PP development is initiated in *Sharpin^cpdm^* mutant mice, essential compartmentalization which normally begins prior to the entry of lymphocytes fails. Efficient segregation into B and T cells areas is lacking, and the previously described absence of FDCs, possibly a consequence of the faulty B cell maturation or defects in stromal FDC precursors, leads to absence of B cell follicles [36], [37]. Changes in the spleen, although less severe, parallel those observed in the PPs. Increased extramedullary myelopoiesis accompanies accumulation of granulocytes in the spleens of mutant mice over time. Whereas organization, compartmentalization, and lymphocyte accumulations increase with maturity in spleens of controls, a more primitive white pulp structure is evident in mutant mice, with increased mixing of B and T cells beginning between 2 and 4 weeks of age, and failure of these compartments to expand.

Cooperative signaling between stromal tissues and hematopoetic cells regulates cell survival, differentiation, and proliferation, as well as the segregation and trafficking of B and T lymphocytes in secondary lymphoid organs during development and in immune responses. Loss of one or more of these signals likely leads to the lack of immunologically competent compartments and eventual regression of PP in *Sharpin^cpdm^* mutant mice.

*Sharpin* is conserved among several species, including mice and humans [38]. Such evolutionary conservation suggests a biological advantage. Results of this and other studies suggest that SHARPIN has essential roles in secondary lymphoid organ development, maturation of B cells and subsequent immunoglobulin production, and control of inflammation. At least some of the developmental abnormalities observed in *Sharpin^cpdm^* mutant mice in the present study appear to be caused by the absence of SHARPIN in stromal cells, because the compartmentalization of PP stromal cells is independent of lymphocyte entry [3]. Furthermore, irradiation of juvenile *Sharpin^cpdm^* mutant mice and transplantation with bone marrow from healthy donors at a time when PP are present did not prevent complete regression of PP by 8 weeks of age.

Despite a lack of normal organization, lymphocytes were recruited into PP anlagen in neonatal mutant mice, indicating that SHARPIN is essential for the organization and maintenance, but not the initial development of PP.

## Methods

### Mice

All experiments involving mice (breeding, genotyping, bone marrow transplantation, and euthanasia) followed procedures approved by The Jackson Laboratory Animal Care and Use Committee under ACUC approvals 07005 and LDS 02–03.

### Evaluation of PP Development in Juvenile Mice

Mice used for IFA, immunohistochemistry, and WMI evaluations were F2 and F3 offspring from a cross between *Sharpin^cpdm^* mutant males and wildtype females of a congenic strain (C.CAST-(*D15Mit156-D15Mit2*)/Sun), created for fine mapping of the *Sharpin* gene [39], [40]. Mice carrying C57BL/KaLawRij*-Sharpin^cpdm^*/RijSunJ alleles at markers flanking the *Sharpin* region were identified as mutants. Mice carrying CAST/EiJ alleles, or heterozygotes, were identified as controls. At least 3 mice per group were examined for each time point. Although no gross effect on the phenotype was observed in the mapping cross, to verify that the phenomenon of PP regression was not attributable to background genetics of the congenic mapping cross, we examined sections of the small intestine of C57BL/KaLawRij*-Sharpin^cpdm^*/RijSunJ mice at 4 to 7 weeks of age. Results were similar to those seen in mice from the congenic mapping cross (data not shown).

WMI was performed as previously described with minor modifications [3], [41], using antibodies to ICAM1 (biotinylated, Pharmingen, 3E2) or B220 (biotinylated, Pharmingen C363.16A). Briefly, tissues collected at necropsy were immediately fixed in 4% paraformaldehyde on ice. After 30 minutes, tissues were transferred into 4% glycine on ice for 30 minutes, then gradually dehydrated in methanol (MeOH). Blocking was done in 30% H_2_O_2_ in MeOH for 30 minutes, followed by blocking in PBS with 2% dry milk powder and 0.1% Triton-X 100 (PBMST) twice for 1 hour at RT. Biotinylated primary antibody was applied at 2 ug/mL in PBMST overnight at 4C. After 5 washes in PBMST at 4C, antibodies were detected using ABC kit (Vectastain Elite, Vector Labs) at a 1∶20 dilution in PBS, for 2 hours at 4C. After 5 washes in PBMST at 4C, and a final wash in PBST at RT, diaminobenzidine solution was applied at 250 mg/mL (with 0.05% CoCl_2_ in some assays) until a color change was observed, followed by copious washing in PBST. Tissues were gradually dehydrated in MeOH and examined and photographed using an Olympus SZX12 dissecting microscope and Olympus DP70 digital camera. Counts of ICAM1^+^ patches were analyzed for significance by Student’s *t*-test and Welch’s *t*-test.

For histologic evaluation of cross-sections, Swiss rolls of small intestine collected at necropsy were prepared using a modification of methods previously described [42]. Paraffin-embedded, Fekete’s acid alcohol formalin fixed tissues were sectioned at 5 µm and stained with H&E. For immunofluorescence, a 5-cm segment of small intestine proximal to the cecum in mice >2 weeks of age, or Swiss rolls of the small intestine in younger mice, and spleens were prepared in Optimal Cutting Temperature (OCT, 4583, Sakura, Torrance CA, USA) compound. The small intestine was removed at necropsy into PBS on ice, then infused with a mixture of OCT diluted with 5% sucrose in PBS prior to embedding in OCT and freezing on dry ice. Blocks were stored at −80C until use, sectioned at 8 µm and collected on Superfrost Plus glass slides (Fisher, Rockville, IL, USA). Slides were fixed in acetone at −20C for 10 minutes and air dried at room temperature for 15 minutes before washing in PBS, and then placed in an incubation/humidity chamber. Tissue was incubated with hamster anti-mouse CD3e (clone 500A2, Pharmingen, NJ, USA) and rat anti-mouse CD45RB (protein tyrosine phosphatase, receptor type, C, isoform B; clone 16A, Pharmingen) with 3% FBS 2 hours at room temperature. Slides were washed in PBS and incubated with secondary antibody (AlexaFluor 488 goat anti-rat IgG, AlexaFluor 546 goat anti-hamster IgG, Molecular Probes, Invitrogen, Carlsbad, CA, USA) in PBS and then counterstained with 4′,6-Diamidino-2-phenylindole, dialactate (DAPI; D9564, Sigma, St. Louis, MO, USA). Slides were washed in PBS, cover slipped with Fluoromount mounting medium (F4680, Sigma), and examined and photographed using a Leica DMRXE microscope and DFC300FX camera or Leica SP5 AOBS confocal microscope.

### Bone Marrow Transplantation

Donor mice for bone marrow transplantation were healthy adult homozygous C57BL/6-Tg(UBC-GFP)30Scha/J females. Recipient mice were 7-day-old C57BL/KaLawRij*-Sharpin^cpdm^*/RijSunJ mutant and control (heterozygous or wildtype; C57BL/KaLawRij*-Sharpin^cpdm^/+* or +/+, respectively) mice. Prior to transplantation, histocompatibility of donor and recipient mouse strains was confirmed by maintenance of full thickness dorsal skin grafts as described elsewhere [43]. Genotypes of recipient mice were identified via pyrosequencing prior to transplantation.

Femurs and tibias of donor C57BL/6-Tg(UBC-GFP)30Scha/J mice were surgically removed with sterile forceps and scissors immediately after euthanasia by CO_2_ asphyxiation. Bones were placed in a sterile petri dish containing 5 mL of sterile Hanks Buffered Saline Solution (HBSS) with 10% FBS. A sterile mortar and pestle were used to gently crush the bones, and material was placed in a sterile 50 mL tube and vortexed to release the cells and allow bone fragments settle to the bottom of the tube. The supernatant was decanted and poured through a 70 µm mesh strainer; 10 mL of HBSS was added to the filtered supernatant and the process was repeated once. Cells were centrifuged, the supernatant discarded, and red blood cells were lysed with 10 mL of ammonium chloride lysis buffer (0.15M NH_4_Cl, 10 mM KHCO3, 0.1 mM Na_2_EDTA, pH 7.4) on ice for 10 minutes. The remaining cells were rinsed twice with HBSS (1 to 5 mL) and viable cells were counted with a hemacytometer.

Recipient *Sharpin^cpdm^* (n = 8; 4 females and 4 males) and control mice (8; 5 females and 3 males) were irradiated at 500 cGy by use of a Cs irradiator as previously described [44]. Mice were then injected intraperitoneally with 1×10^7^ prepared bone marrow cells from homozygous GFP^+^ donors. Mice were observed daily for one week following engraftment and twice weekly from then on. By 1.5 weeks of age hair loss was evident and at 3 weeks of age mice were completely hairless. Hair regrowth was detected at 4 weeks of age and appeared complete by 6 weeks of age.

Two male *Sharpin^cpdm^* mice died at 8 weeks of age and tissues were not available for necropsy. Following euthanasia at 7 to 8 weeks of age, complete necropsies were performed in the remaining 6 *Sharpin^cpdm^* (4 female and 2 male) and 8 control mice (5 female and 3 male). Tissues were collected at necropsy and placed in Fekete’s acid alcohol formalin solution. Slides were prepared from paraffin-embedded tissues (5 µm thick sections, including Swiss rolls of the intestine), routinely stained with H&E, and read by a veterinary pathologist (JPS). The pathologist was not informed of identification and genotype of the mice at the time of evaluation; however, skin lesions considered typical of the *Sharpin^cpdm^* phenotype were evident to some degree in all mutant mice, thus evaluation could not be performed in a completely blinded manner. Engraftment was confirmed in all recipients by detection of GFP^+^ cells as previously described [7] in the spleen, liver, colon, and cecum.
